# A useful technique to maintain the position of the hand following abdominal flap cover

**DOI:** 10.4103/0970-0358.41128

**Published:** 2008

**Authors:** Hari Venkatramani, S. Raja Sabapathy

**Affiliations:** Department of Plastic, Hand and Reconstructive Microsurgery, Ganga Hospital, Coimbatore, Tamil Nadu, India

Dear Sir,

Pedicled abdominal flaps are commonly performed to provide soft tissue cover for post-traumatic defects in the hand and upper limb. Following flap transfer, the hand has to be positioned well to prevent kinking of the pedicle. In the immediate postoperative period, this is done with the help of adhesive plasters and sometimes with flap-positioning splints. Later, the patient and the relatives are instructed about the correct position. Whichever technique is used, it would be good to have a system whereby the position preferred by the surgeon is conveyed to the people who monitor the flap in the ward and at home in a simple and straightforward manner, which is easy to understand and replicate. We have found the following technique to be very useful over the last four years.

**Figures 1 and 2:**
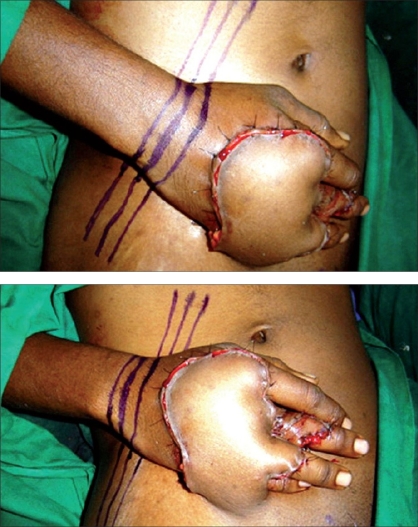
The lines to monitor the position of the flap

After the flap is inset into it's final position, the hand is held in the most optimal position and three parallel lines are drawn with marking ink just proximal to the wrist. The lines start from the abdominal wall proximally, then across the distal forearm and continue distally again over the abdominal wall [Figures 1 and 2]. The patient is shifted from the operating table after the lines are drawn. During the transfer of the patient and immediately after the transfer to the bed in the ward, the position is again checked - the lines must be in continuity. In the ward, the significance of the lines and the need to prevent kinking of the pedicle are explained to the staff nurse and the relatives of the patient. Most of these patients are discharged between seven and ten days after the operation and readmitted for flap division. During the period of home stay, these lines serve as a ready and easy guide to the relatives to maintain the correct position of the limb.

We recommend this easy technique of monitoring flap positioning to prevent kinking of the pedicle.

